# Experimental and Theoretical Studies of Novel Azo Benzene Functionalized Conjugated Polymers: *In-vitro* Antileishmanial Activity and Bioimaging

**DOI:** 10.1038/s41598-019-56975-x

**Published:** 2020-01-09

**Authors:** Neetika Singh, Mohd. Arish, Prabhat Kumar, Abdur Rub, Ufana Riaz

**Affiliations:** 1 Materials Research Laboratory Department of Chemistry, Jamia Millia Islamia, New Delhi, 110025 India; 2 Department of Biotechnology, Jamia Millia Islamia, New Delhi, 110025 India; 3 Advance Instrumentation Research Facility, Jawaharlal Nehru University, New Delhi, 110067 India

**Keywords:** Materials chemistry, Theoretical chemistry

## Abstract

To study the effect of insertion of azobenzene moiety on the spectral, morphological and fluorescence properties of conventional conducting polymers, the present work reports ultrasound-assisted polymerization of azobenzene with aniline, 1-naphthylamine, luminol and o-phenylenediamine. The chemical structure and polymerization was established via Fourier transform infrared (FTIR) spectroscopy, nuclear magnetic resonance (^1^H-NMR) spectroscopy, while the electronic properties were explored via ultraviolet-visible (UV-vis) spectroscopy. Theoretical IR and UV spectra were computed using DFT/B3LYP method with 6–311G basis set while theoretical ^1^H-NMR spectra was obtained by gauge independent atomic orbital (GIAO) method. The theoretically computed spectra were found to be in close agreement with the experimental findings confirming the chemical as well as electronic structure of the synthesized polymers. Morphology was investigated by X-ray diffraction and transmission electron microscopy studies. Fluorescence studies revealed emission ranging between 530–570 nm. The polymers also revealed high singlet oxygen (^1^O_2_) generation characteristics. *In-vitro* antileishmanial efficacy as well as live cell imaging investigations reflected the potential application of these polymers in the treatment of leishmaniasis and its diagnosis.

## Introduction

Leishmaniasis is one of the most neglected tropical diseases caused by parasite belonging to genus *Leishmania*^1^. Although leishmaniasis encompasses different forms of clinical manifestations, the most severe form is visceral leishmaniasis (VL) caused by *Leishmania donovani* or *Leishmania infantum*. VL results in anemia, weight loss, enlargement of spleen/liver in patients and can be life-threatening if left untreated. Although there is a substantial decrease in the VL cases reported during the past few decades, the rise in post-kalazar dermal leishmaniasis remains a major hurdle in VL elimination program in the Indian subcontinent as well as worldwide^2^. Current therapy for VL is expensive and associated with several side effects. In addition, rise in drug resistance in parasites against conventional drugs has demanded the development of novel, effective and least cytotoxic alternative agents capable of early detection and diagnosis of leishmaniasis^3,4^.

Azobenzene based polymers play a vital role in optoelectronic applications and are often coupled/functionalized with various conjugated polymers to improve their optical properties^5,6^. The insertion of azobenzene chromophore in the polymer chain via covalent bonding/blending gives rise to a number of unfamiliar effects under visible and ultraviolet irradiations^7,8^. *Smitha et al*.^9^ synthesized azobenzene monomers using substituted phenols, biphenyls and naphthyl rings to obtain nonlinear optical (NLO) polymers. Similarly, *Huang et al*.^10^. designed azobenzene and thymine (T) functionalized conjugated copolymers (PTCAz-T) via Suzuki coupling polymerization and found that the polymers underwent trans-to-cis isomerization upon irradiation with laser light. *Mao et al*.^11^ prepared polymeric waterborne polyurethane containing azobenzene units, while *Izumi et al*.^12^ synthesized poly(phenylenevinylene) based conjugated polymers with azobenzene groups attached with the main chains through Pd-catalyzed coupling polymerization. These polymers are generally photo-active under UV light which limits their biological applications as UV light is strongly scattered by biological systems and also damages the cells and tissues. Hence several strategies have been adopted to address the visible region by designing azo based polymers via energy transfer^13^, electron transfer^14^, push-pull system^15^ and also through extended π-conjugation^16^.

Conjugated polymers such as polyaniline (PANI)^17^, poly(1-naphthylamine) (PNA)^18^, polypyrrole (Ppy)^19^, polythiophene (PTh)^20^, polycarbazole (PCz)^21^ etc. have been widely explored due to their amazing opto-electronic properties. These polymers have been extensively used in the field of biosensors and bio-imaging^22^. Our earlier studies have shown that the fluorescent properties of these polymers can be effectively tailored via effective copolymerization and doping^23–25^. Conjugated copolymers of PNA, poly(o-phenylenediamine) (PPd) and poly(o-anisidine) (PoA) were synthesized via microwave –assisted polymerization and the polymers exhibited molar masses ranging between 6400–8600. The polymers showed intense fluorescence emission around 531 nm which was attributed to the synergistic effect of copolymerization^23^. Likewise, copolymers of o-phenylenediamine with luminol were prepared via ultrasound-assisted polymerization which showed viscosity average molar mass ranging between 10000–12000 while an intense emission in the 600 nm region was attained in the copolymer containing higher number of PPd units^26^. Similarly, doping of PPd with luminol was reported and it exhibited fluorescence in the near infrared (NIR) region which was governed by the chemical structure of luminol under acidic, basic and neutral conditions^22^. The molecular weight was calculated to be ranging between 12,328–21,528. PPd and its dye based adducts were successfully synthesized using different dyes such as Acid Orange (AO), fluorescein (Fluo) and Rhodamine 6G (R6G)^27^. Doping was established by UV-visible and XPS studies. The molecular weight was determined by Gel Permeation Chromatography (GPC) technique using 0.03% polymer solution dissolved in n-methyl-2-pyrrolidone (NMP) containing 0.1% LiCl and the weight average molecular weights (M_w_) were found to be ranging between 10,500–19,000^23,27^.

Polymers containing azobenzene moieties exibit remarkable opto-electronic due to the presence of higher extent of conjugation^28^. The redox state of these polymers can be easily controlled with the monomer functionality which can help in tuning the fluorescence emission in the desired range. Thus with the aim to study the influence of azobenzene insertion on the optoelectronic properties of amine functionalized conjugated polymers, the present work reports the ultrasound-assisted chemical polymerization of aniline, 1-naphthylamine, luminol and o-phenylenediamine with benzene diazonium chloride. The above-mentioned monomers were chosen in particular as they show polymerization via NH linkage. The synthesized polymers were characterized via fourier transform infrared spectroscopy (FTIR), nuclear magnetic resonance spectroscopy (^1^H-NMR), ultraviolet-visible spectroscopy (UV-vis) while the morphology was investigated by using X-ray diffraction (XRD) and transmission electron microscopy (TEM) studies. The DFT calculations were performed using BLY3P/6311G basis set. The theoretical IR, ^1^H-NMR, UV spectra were computed and compared with the experimentally observed data. *In-vitro* anti-leishmanial efficacy and cytotoxicity of the polymers were evaluated. Live cell imaging studies were also carried out to explore the extent of penetration as well as binding of the polymers with the *Leishmania*-promastigotes.

## Experimental

o-phenylenediamine (C_6_H_8_N_2_, Sigma Aldrich, USA), luminol (Sigma Aldrich, USA), Aniline (C_6_H_7_N Fisher Scientific), 1-naphthylamine (Loba Chemie), ferric chloride (FeCl_3_·6H_2_O, Merck India), N-methyl-2-pyrrolidone (NMP) (Merck, India), ethanol (Merck, India), dimethyl formamide (DMF) (S.d. Fine Chem., Pvt. Ltd, India) Sodium Nitrite (Merck, India), hydrochloric acid (HCl) (Merck, India) were used without further purification.

### Synthesis of the precursor benzene diazonium salt

Sodium nitrite (5 g) was added to 250 ml Erlenmeyer flask containing deionized water (50 ml). Hydrochloric acid (35%, 30 ml) was added to the above mixture and was cooled in an ice-bath. The obtained color of the reaction mixture was blackish red which was isolated by freeze-drying. The mass was recrystallized from ethanol-water mixture to provide orange-red crystals. The solution was then cooled below 0 °C and freeze dried. The obtained powder was refrigerated at 10 °C.

### Synthesis of azo benzene based conjugated polymers

**Scheme 1:**
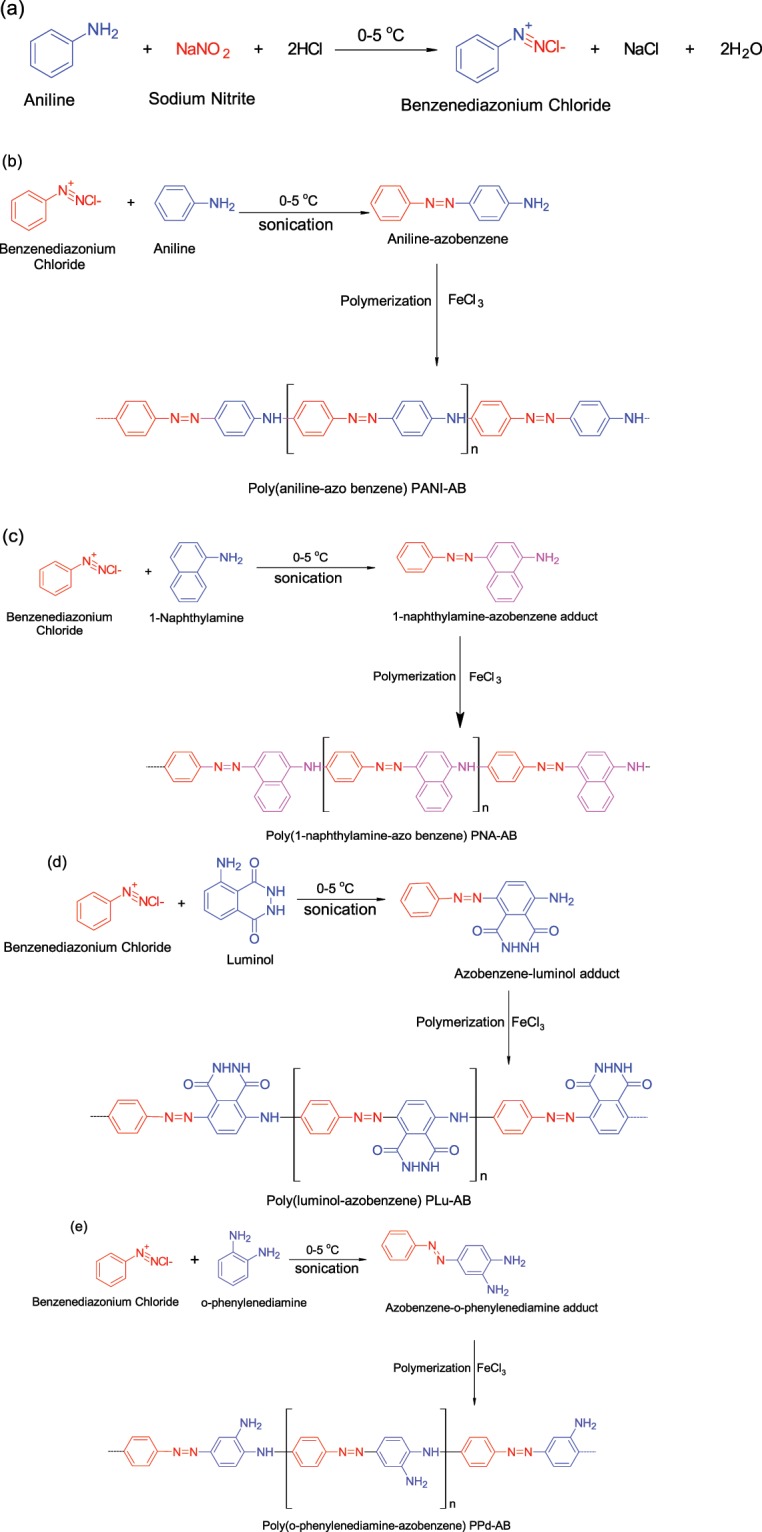
(**a**) Synthesis of benzenediazonium chloride and mechanism of polymerization of (**b**) PANI-AB, (**c**) PNA-AB, (**d**) PLu-AB, (**e**) PPd-AB.

Aniline (40 ml, 4.3 × 10^−1^ mol) was added to an Erlenmeyer flask (250 ml) containing the synthesized diazonium salt (1.4057 g, 1 × 10^−2^ mol). The reaction mixture was sonicated at 0–5 °C and after an interval of 15 min, ferric chloride (1 g, 3.8 × 10^−3^ mol) was added to the above reaction mixture which changed from pale yellow to dark green. The reaction was further carried out for 5 h at the same temperature. The obtained polymer was then kept in a deep freezer for 24 h at −5 °C and was centrifuged, washed several times with distilled water on a R-8 C laboratory centrifuge and then dried in a vacuum oven for 24 h at 70 °C to ensure complete removal of solvent and other impurities. The synthesized polymers were designated as poly (aniline-azobenzene) (PANI-AB), poly(1-naphthylamine-azobenzene) (PNA-AB), poly (luminol-azobenzene) (PLu-AB), and poly (o-phenylenediamine-azobenzene) (PPd-AB) as shown in Scheme 1(a–e). The intrinsic viscosity was meaured as per method reported in our previous studies^23^. The values of intrinsic viscosity were calculated to be 0.41 for PANI-AB, 0.45 for PNA-AB, 0.42 for PLu-AB and 0.47 for PPd-AB. Hence, the weight average molecular weights (M_w_) were presumed to be ranging between 10,500–19,000^27^.

## Characterization

### Spectral analysis

FT-IR spectra of conjugated polymers were taken on FT-IR spectrophotometer (Shimadzu, Model IRA Affinity-1 while Ultraviolet-visible light (UV-vis) spectra were recorded on UV-vis spectrophotometer model Shimadzu, UV-1800 using CHCl_3_ as solvent. ^1^H-NMR spectra were recorded at 25 °C on a Bruker 300 MHz spectrometer using deuterated CHCl_3_. Fluorescence studies were performed on fluorescence spectrophotometer Fluorolog@ 3–11 using NMP solvent.

### Morphological analysis

XRD patterns of the conjugated polymers were recorded on Philips, Model PW 3710 using Ni filtered Cu-Kα radiation). TEM micrographs (TEM) were obtained using Morgagni Model 268-D TEM system (FEI, USA).

### Theoretical studies

The electronic properties (UV-Vis calculations) of the polymers, such as HOMO-LUMO energies, absorption wavelengths and oscillator strengths were performed by using TD-DFT/B3LYP method with 6–311G basis set. The ^1^H-NMR spectra were computed using the gauge independent atomic orbital (GIAO) method.

### THP-1 culture, parasite culture and anti-leishmanial activity

The THP-1 monocyte cell line was maintained in RPMI 1640 medium (Life Technologies) supplemented with 10% heat-inactivated FBS (Life Technologies) and 1% streptomycin-penicillin (Life Technologies) at 37 °C in 5% CO_2_. The differentiation of THP-1 monocytes into macrophages was performed by incubating THP-1 with 5 ng/ml PMA (phorbol 12-myristate 13-acetate; Sigma) at 37 °C in 5% CO_2_ for 24 h. *Leishmania donovani* (MHOM/IN/1980/AG83) promastigotes were grown in M199 medium (Gibco) supplemented with 10% heat-inactivated fetal bovine serum (FBS) (Gibco) and 1% Penicillin-streptomycin solution (Gibco) at 22 °C. Promastigotes from late log phase at a density of 2 × 10^6^ cells/ml were incubated in the absence and or presence of PANI-AB, PNA-AB, PLu-AB and PPd-AB (10 μg/ml) for 48 h at 22 °C. The parasites were fixed in 1% paraformaldehyde and counted on a hemocytometer by using an inverted microscope.

### Growth reversibility and MTT assay

*Leishmania donovani* promastigotes untreated and treated with PANI-AB, PNA-AB, PLu-AB and PPd-AB (10 μg/ml) after 7 days were washed and resuspended in fresh media for the next 96 h. The parasites were fixed in 1% p-formaldehyde and counted on a hemocytometer using an inverted microscope. For the MTT assay, 2 × 10^5^ THP-1 monocytic cell line per well were seeded onto a 96 well-plate and were treated with PMA for differentiation into macrophages for 24 h. Next day, the cells were washed, and the media was replaced with fresh media and were cultured for an additional 24 h in the presence of compounds PANI-AB, PNA-AB, PLu-AB and PPd-AB (100–6.25 μg/mL). Cell viability was determined using the MTT cell viability assay. 3-(4,5-Dimethyl-2-thiazolyl)-2,5-diphenyl-2H-tetrazolium bromide, MTT (Sigma-Aldrich), was applied in the dark, following a 4 h incubation at 37 °C. The MTT-containing medium was replaced with 200 μL of isopropanol-HCl (0.1 N) and kept at 37 °C for 10 min to solubilize the formazan crystals. The samples were transferred to 96-well plates, and the absorbance of the converted dye was measured at 570 nm. The cell viability of the control (non-treated) cells was taken as 100%. *In-vitro* antileishmanial and cytotoxic activities were expressed as IC50 and CC50, respectively by the linear regression analysis.

## Results and Discussion

### Geometry optimization and distribution of charges as well as frontier molecular orbitals

The optimized structures of AB, PANI-AB, PNA-AB, PLu-AB and PPd-AB are given in, Fig. S1(a–e). The C-C and C=C bond lengths for AB molecule were noticed to be 1.39 Å and 1.38 Å respectively, whereas the C-N bond length was found to be 1.42 Å. The N=N bond length was found to be 1.07 Å. The bond lengths for optimized structure of PANI-AB were computed to be 1.40 Å and 1.35 Å respectively, while the C-N and N=N bond lengths were found to be 1.47 Å and 1.23 Å respectively. The N-H bond length was computed to be 0.998 Å. The structure was observed to be planar. Similarly, the optimized geometry of PNA-AB revealed the C-C and C=C bond lengths to be 1.42 Å and 1.35 Å respectively, while the C-N and azo bond lengths were observed to be 1.41 Å and 1.24 Å respectively. The N-H bond length was found to be 0.998 Å. The C-C and C=C bond lengths for PLu-AB were computed to be 1.39 Å and 1.35 Å respectively and the C-N, C=O, N-H and azo bond lengths were found to be 1.47 Å, 1.26 Å, 0.998 Å and 1.23 Å respectively. A slight reduction was noticed in the C-C and C=C bond lengths of this polymer as compared to the previous ones. The structure however was found to be planar. Interestingly, PPd-AB exhibited a highly twisted configuration and the C-C, C=C bond lengths were computed to be 1.38 Å and 1.37 Å respectively. The C-N bond length was observed to be 1.41 Å while the N-H bond length was computed to be 0.996 Å.

**Figure 1:**
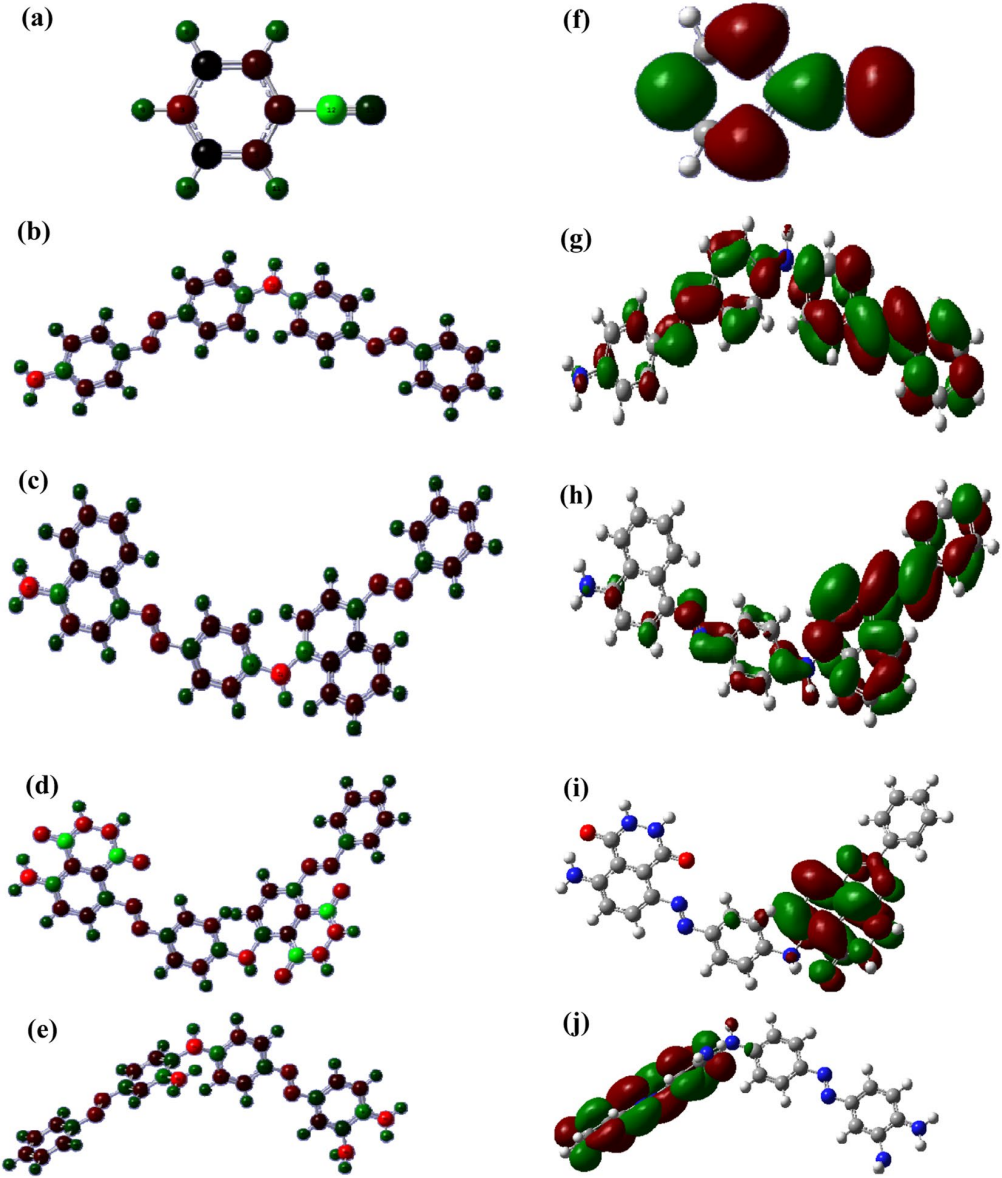
Mulliken charge distribution in (**a**) AB, (**b**) PANI-AB, (**c**) PNA-AB, (**d**) PLu-AB, (**e**) PPd-AB, frontier HOMO-LUMO orbitals in (**f**) AB, (**g**) PANI-AB, (**h**) PNA-AB, (**i**) PLu-AB, (**j**) PPd-AB.

The charge distribution and HOMO-LUMO orbitals in AB and its polymers are shown in Fig. 1(a–j). AB molecule showed charge concentration mainly around carbon atoms, Fig. 1(a), while in case of PANI-AB, the charge was found to be concentrated around the N-H bonds, Fig. 1(b). The charge distribution in PNA-AB, Fig. 1(c), and PPd-AB, Fig. 1(e), was found to be quite similar to that of PANI-AB whereas PLu-AB, Fig. 1(d), exhibited higher negative charge distribution on the carbon atoms adjacent to the C=O and NH linkages. The HOMO and LUMO orbitals were noticed to be highly delocalized in case of AB molecule, Fig. 1(f), as well as in AB modified polymers, Fig. 1(g–j) thereby confirming higher extent of spatial overlap between the two orbitals. This would result in the reduction of band gap, thereby facilitating charge injection of electrons into LUMO or holes into HOMO orbitals. Hence, hybridization of the conducting polymer chains upon insertion of AB was observed to reduce the band gap value as shown in Table 1. The reduction in HUMO–LUMO separation results in the formation of oligomers/polymers with near infrared (NIR) absorption characteristics. The HOMO-LUMO band gap for AB molecule was calculated to be 10.77 eV, Table 1, while it was found to be 5.50 eV for PANI-AB, 4.47 eV for PNA-AB, 3.89 eV for PLu-AB and 4.89 eV for PPd-AB. The band gap was found to be lowest for PLu-AB due to the presence of carbonyl linkages that facilitate the charge transfer through the polymeric chain.

**Table 1:** HOMOs, LUMOs, band gap energies, and quantum yield values of AB, PANI-AB, PNA-AB, PLu-AB and PPd –AB.

Sample	HOMO (eV)	LUMO (eV)	Band gap (eV)	λ_max_ (UV absorption)		Oscillator strength		λ_em_ (fluorescence)	Quantum yield (ϕ)
				Exp.	Theor.	Exp.	Theor.		
AB	−14.56	−3.79	10.77	285	290	0.25	0.26	—	—
PANI-AB	−7.29	−1.74	5.50	400	400	0.83	0.85	570	0.68
PNA-AB	−6.46	−1.99	4.47	450	460	0.84	0.85	530	0.64
PLu-AB	−5.20	−1.25	3.95	280	270	1.3	1.6	530	0.66
PPd-AB	−6.98	−2.07	4.89	370	400	0.32	0.34	570	0.69

### Theoretical and experimental spectral studies of AB, PANI-AB, PNA-AB, PLu-AB and PPd-AB

The experimental IR spectrum of azobenzene (AB) (given in Supplementary Information as Fig. S2(a)), Table 2 revealed peaks at 3498 cm^−1^ and 3477 cm^−1^ due to the OH stretching vibrations while the theoretical data revealed the same peak centred at 3367 cm^−1^. The peak for N_2_ stretching vibrations was observed at 2434 cm^−1^ and the theoretical spectrum revealed the same vibrations at 2416 cm^−1^. The peaks at 1546 cm^−1^, 1535 cm^−1^ and 1529 cm^−1^ were attributed to quinonoid ring stretching vibrations, while the peaks at 1510 cm^−1^, 1504 cm^−1^, 1344 cm^−1^, 1325 cm^−1^ and 1274 cm^−1^ were correlated to benzenoid ring stretching vibrations. Similar peaks were observed in the theoretical spectrum. The CN stretching vibrations were observed at 1230 cm^−1^ and 1201 cm^−1^ while the aromatic ring stretching vibrations were noticed at 835 cm^−1^, 781 cm^−1^, 752 cm^−1^, 692 cm^−1^, 686 cm^−1^, 594 cm^−1^ and 572 cm^−1^. The spectrum of PANI-AB (given in Supplementary Information as Fig. S2(b)) exhibited peaks due to NH stretching vibration at 3441 cm^−1^, 3417 cm^−1^ and 3140 cm^−1^, while the theoretical spectrum revealed peaks at 3168 cm^−1^, 3152 cm^−1^ and 3120 cm^−1^. The azo stretching vibrations were noticed around 2455 cm^−1^, 2160 cm^−1^ and 1984 cm^−1^. The theoretical spectrum also revealed peaks in the same region.

**Table 2:** Theoretical and experimental IR data of PANI-AB, PNA-AB, PLu-AB and PPd-AB.

Sample	Functional Group	Peak Position (cm^−1^)	
		Experimental	Theoretical
Benzene diazonium salt	O-H stretching	3498, 3477	3367
	N=N stretching	2434	2416
	C=C stretching (quinonoid)	1546, 1535, 1529	1596, 1582, 1575
	C=C stretching (benzenoid)	1510, 1504, 1467, 1344, 1325, 1274	1504, 1463, 1456, 1351, 1344, 1288
	C-N stretching	1230, 1201	1232, 1218
	Substituted aromatic ring	835, 781, 752, 692, 686, 594, 572	840, 820, 707, 693, 686, 581, 574
PANI-AB	N-H/O-H stretching	3441, 3417, 3140	3168, 3152, 3120
	N=N stretching	2455, 2160, 1984	2456, 2160, 1984
	Imine stretching	1660, 1610	1656, 1648, 1608
	C=C stretching (quinonoid)	1548, 1531	1552
	C=C stretching (benzenoid)	1469, 1410, 1346, 1334	1504, 1456, 1432, 1352, 1344
	C-N stretching	1232, 1140, 1080	1216, 1200, 1192, 1184, 1136, 1076
	Substituted aromatic ring	887, 867, 798, 785, 732, 720, 690	880, 872, 856, 768, 760, 720, 696
PNA-AB	N-H/O-H stretching	3120, 3020	3181, 3127
	N=N (azo group)	2206, 2175, 2166	2205, 2160, 2156
	Imine stretching	1641	1650
	C=C stretching (quinonoid)	1568	1570
	C-H stretching (benzenoid)	1502, 1485, 1469, 1332, 1292,	1507, 1471, 1462, 1381, 1309, 1287
	C-N stretching	1259, 1207, 1166, 1066	1265, 1201, 1147, 1030
	Substituted aromatic ring	929, 910, 893, 850, 827, 794, 781, 758, 711	967, 904, 895, 868, 823, 796, 778, 760, 715
PLu-AB	N-H/O-H stretching	3410, 3020	3410, 3176
	N=N (azo group)	2164, 2156	2168, 2152
	Imine stretching	1639, 1631, 1600	1632, 1608, 1600
	C=C stretching (quinonoid)	1546, 1541, 1535, 1529	1544, 1540, 1536, 1528
	C=C stretching (benzenoid)	1467, 1462, 1440, 1359, 1342, 1307	1464, 1456, 1440, 1352, 1344, 1304
	C-N stretching	1251, 1230, 1205	1248, 1216, 1208
	Substituted aromatic ring	964, 927, 896, 829, 796, 777, 725, 657, 644, 628, 588	952, 928, 888, 824, 792, 776, 720, 656, 648, 632, 576
PPd-AB	N-H/O-H stretching	3210, 3035, 3030	3361, 3352, 3025
	N=N (azo group)	2272, 2233, 2167	2268, 2220, 2156
	Imine stretching	1610	1612
	C=C stretching (quinonoid)	1552, 1546	1550, 1545
	C=C stretching (benzenoid)	1492, 1467, 1344, 1327, 1274	1480, 1458, 1336, 1327, 1273
	C-N stretching	1246, 1230	1237, 1228
	Substituted aromatic ring	974, 933, 885, 866, 798, 781, 653, 638, 617	814, 778, 769, 760, 751, 661, 652, 634, 616

The imine stretching vibrations were observed around 1660 cm^−1^ and 1610 cm^−1^ whereas quinonoid as well as benzenoid ring stretching vibrations were noticed at 1548 cm^−1^, 1531 cm^−1^, 1469 cm^−1^, 1410 cm^−1^, 1346 cm^−1^ and 1334 cm^−1^ respectively. The CN stretching vibrations were found at 1232 cm^−1^, 1140 cm^−1^ and 1080 cm^−1^ while the substituted aromatic ring vibrations were noticed at 887 cm^−1^, 867 cm^−1^, 798 cm^−1^, 785 cm^−1^, 732 cm^−1^, 720 cm^−1^ and 690 cm^−1^. The IR spectrum of PNA-AB (given in Supplementary Information as Fig. S2(c)) revealed NH stretching vibration peaks around 3120 cm^−1^ and 3020 cm^−1^ whereas the N_2_ stretching vibrations were found at 2206 cm^−1^, 2175 cm^−1^ and 2166 cm^−1^. The peaks showed a considerable shift as compared to PANI-AB. The imine stretching peaks were noticed at 1641 cm^−1^ whereas the quinonoid and benzenoid ring stretching peaks were noticed at 1568 cm^−1^, 1502 cm^−1^, 1485 cm^−1^, 1469 cm^−1^, 1332 cm^−1^ and 1292 cm^−1^ respectively^18^, which were similar to the peaks observed in PANI-AB. Multiple CN stretching vibration peaks were observed at 1259 cm^−1^, 1207 cm^−1^, 1166 cm^−1^ and 1066 cm^−1^ whereas the substituted aromatic ring vibration peaks were noticed at 929 cm^−1^, 910 cm^−1^, 893 cm^−1^, 850 cm^−1^, 827 cm^−1^, 794 cm^−1^, 781 cm^−1^, 758 cm^−1^ and 711 cm^−1^. Likewise, the IR spectrum of PLu-AB (given in Supplementary Information as Fig. S2(d)) revealed peaks at 3410 cm^−1^ and 3020 cm^−1^ correlated to NH/OH ring stretching vibrations while the peaks associated with azo group were observed at 2164 cm^−1^ and 2156 cm^−1^ ^22,26^. Multiple imine stretching peaks were noticed around 1639 cm^−1^, 1631 cm^−1^, 1600 cm^−1^ while the qiunonoid benzenoid ring stretching, CN and substituted aromatic vibrations were observed to be similar to the ones found in PANI-AB and PNA-AB. The theoretical data was found to be in close agreement with the experimental one. The spectrum of PPd-AB (given in Supplementary Information as Fig. S2(e)) showed peaks at 3210 cm^−1^, 3035 cm^−1^ and 3030 cm^−1^ associated with the NH stretching vibrations of PPd and CH stretching vibrations of AB while the peaks at 2272 cm^−1^, 2233 cm^−1^ and 2167 cm^−1^ were correlated to the N_2_ stretching vibrations. The imine stretching and quinonoid, benzenoid stretching peaks were found at 1610 cm^−1^, 1552 cm^−1^, 1546 cm^−1^, 1492 cm^−1^, 1467 cm^−1^, 1344 cm^−1^, 1327 cm^−1^ and 1274 cm^−1^ respectively while the CN stretching vibrations were noticed around 1246 cm^−1^ and 1230 cm^−1^ ^26,27^. The substituted aromatic ring vibrations were found at 974 cm^−1^, 933 cm^−1^, 885 cm^−1^, 866 cm^−1^, 798 cm^−1^, 781 cm^−1^, 653 cm^−1^, 638 cm^−1^, and 617 cm^−1^. It can therefore be concluded that the presence of the peaks associated with AB, PANI, PNA, PLu and PPd confirmed the polymerization as well as insertion azo benzene moiety. The presence of multiple NH stretching peaks, N_2_ stretching peaks, CN vibration peaks as well as quinonoid/benzenoid peaks clearly confirmed the polymerization and successful insertion of AB in these polymers. The theoretical spectra of the polymers also revealed peaks in the same region thereby further confirming the proposed structures as well as insertion of AB in the polymers.

The ^1^H-NMR spectrum of AB molecule (Given in Supplementary Information as Fig. S3(a)), Table 3 revealed peaks at δ = 7.27 ppm, 7.6 ppm and 8.2 ppm attributed to the aromatic protons of the benzene ring. Similar protons were also observed in the theoretical spectrum. The ^1^H-NMR spectrum of PANI-AB (Given in Supplementary Information as Fig. S3(b)), Table 3, revealed a peak at δ = 4.4 ppm correlated to the protons of the amine group, while the proton associated with the NH linkage was observed at δ = 5.3 ppm. The theoretically computed ^1^H-NMR spectrum of the same polymer showed protons of the amine group at δ = 4.51 ppm, 4.49 ppm while the protons of the NH linkage were noticed at δ = 5 ppm and 5.37 ppm. The protons associated with AB were noticed around δ = 7.3 ppm, 7.6 ppm and 7.8 ppm. The theoretical spectrum revealed the same protons around δ = 6.46 ppm and 6.56 ppm. The protons of the aniline ring were observed at δ = 6.8 ppm and were found to be in close agreement with the theoretical spectrum. Similarly, the ^1^H-NMR spectrum of PNA-AB (Given in Supplementary Information as Fig. S3(c)), Table 3 showed protons of the amine group around δ = 4.7 ppm, while the theoretical spectrum revealed these protons at δ =4.35 ppm and 4.86 ppm. The protons associated with the benzene ring of 1-napthylmine were observed around δ = 6.7 ppm, 7.4 ppm and 7.8 ppm, while the protons of the azo benzene ring were observed at δ = 7.3 ppm and 7.5 ppm. The theoretical spectrum revealed these protons around δ = 6.53 ppm, 6.79 ppm, 6.81 ppm and 6.86 ppm. The small shift in the values was noticed due to the solvent effect. The ^1^H-NMR spectrum of PLu-AB (Given in Supplementary Information as Fig. S3(d)), Table 3, also revealed protons of the amine group and the NH linkage at δ = 4.7 ppm and 5.6 ppm. The theoretical spectrum of the polymer revealed these protons around δ = 4.35 ppm, 4.86 ppm and 5.0 ppm, 5.4 ppm. The protons of the luminol ring were observed as a broad hump spanning between at δ = 7.4 ppm – 7.9 ppm and the azo benzene protons were noticed at δ = 7.3 ppm. The broad hump obtained in the experimental spectrum was due to the presence of intense hydrogen bonding between the polymer chains. Similar hump was attained in case of PPd-AB (Given in Supplementary Information as Fig. S3(e)), Table 3. The presence of the NH protons as well as the amine protons confirmed the polymerization as well as insertion of AB in PANI, PNA, PLu and PPd. The theoretical spectrum was found to be closely matching with the experimental data which further established the formation of AB incorporated polymers.

**Table 3:** Theoretical and experimental ^1^H-NMR data of PANI-AB, PNA-AB, PLu-AB and PPd- AB.

Polymer	Proton	Chemical shift (ppm)	
		Experimental	Theoretical
Benzene diazonium	Protons of azo benzene	7.2, 7.6, 8.2	7.1, 8.1, 9
PANI-AB	N**H**_**2**_ of aniline ring	4.4	4.51, 4.69
	NH linkage of PANI	5.3	5.0, 5.37
	Protons of azo benzene	7.3, 7.6, 7.8	6.4, 6.56
	Aromatic protons of aniline	6.8	6.6, 6.7
PNA-AB	N**H**_**2**_ attached to 1-naphthylamine	4.8	4.42
	NH of PNA	—	5.85
	Aromatic protons of fused benzene of 1-naphthylamine	6.7, 7.4, 7.8	6.55, 6.69, 6.93, 7.31
	Protons of azo benzene	7.3, 7.5	6.53, 6.79, 6.81, 6.86
PLu-AB	N**H**_**2**_ attached to luminol	4.7	4.35, 4.86
	NH of luminol	5.6	5.0, 5.4
	Aromatic protons of luminol	7.4–7.9	6.56, 6.65,
	Protons of azo benzene	7.3, 7.4–7.9	7.14, 7.22, 7.55
PPd-AB	N**H**_**2**_ of o-phenylenediamine ring	4.3	4.6
	NH of PPd	4.8	5.0, 5.4
	Aromatic protons of o-phenylenediamine	6.9, 7.1	6.0, 6.2, 6.35
	Protons of azo benzene	7.3, 7.5–7.8	6.55, 6.76, 6.9

### Morphological analysis via XRD and TEM

The XRD profile of AB revealed peaks at 2θ = 18.8°, 21.1°, 22.4°, 24.8°, 27.4° and 29.1° (Given in supporting information as Fig. S4). The peak at 21.1° showed maximum intensity confirming higher crystallinity and presence of ordered aromatic chains in this plane. The XRD of PANI-AB revealed reorganization of several planes as compared to the diazonium salt upon incorporation of aniline moiety. The peaks were noticed at 2θ = 19.7°, 20°, 22.1° and 25.1°. The peak at 2θ = 21.1° showed lower intensity while a sharp peak was observed at 2θ = 25.1° corresponding to the plane typically observed in PANI as reported by other authors^29^. Interestingly, the XRD of PNA-AB exhibited several planes at 2θ = 10.60°, 11.61°, 17.07°, 18.28°, 19.4°, 21.3°, 22.52°, 23.73°, 26.56° and 29.2° confirming a highly crystalline and well organized structure. The rigidity and crystallinity were attributed to the presence of fused aromatic ring of PNA. The peak at 2θ = 21.1° appeared to be more pronounced and well formed. The XRD of PLu-AB exhibited an amorphous morphology as a single sharp peak was observed at 2θ = 27°. Similarly the XRD of PPd-AB revealed a broad diffuse hump around 2θ = 21.1° confirming the formation of an amorphous structure. It can therefore be concluded that the incorporation of AB in PANI, PNA, PLu and PPd showed structural reorganization and highly crystalline structure was attained in case of PNA-AB while amorphous structure was achieved in case of PPd-AB.

**Figure 2:**
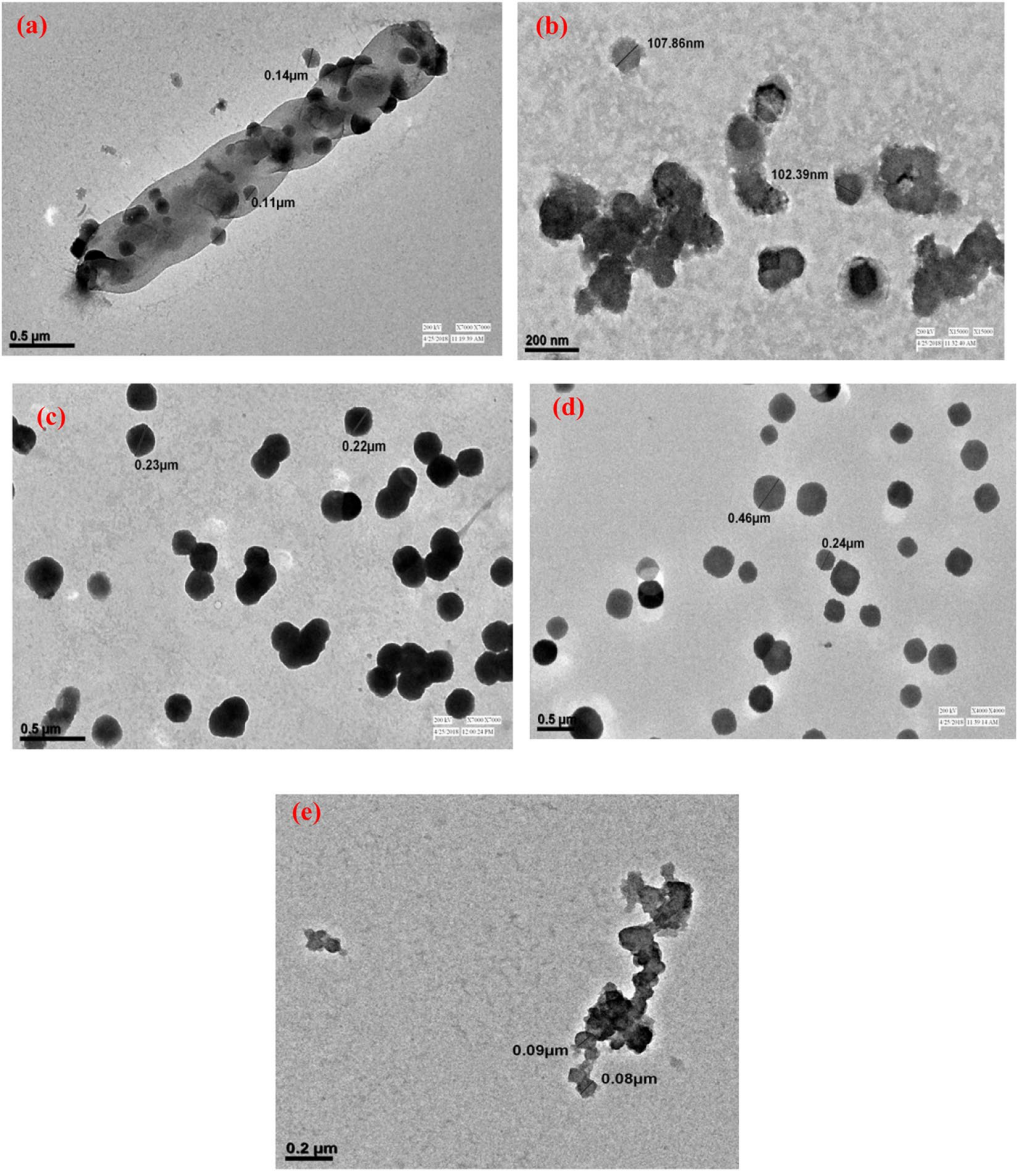
TEM of (**a**) AB, (**b**) PANI-AB, (**c**) PNA-AB, (**d**) PLu-AB, (**e**) PPd-AB.

The TEM of AB Fig. 2(a), showed the formation of a rod like structure containing tiny spherical particles. The TEM of PANI-AB, Fig. 2(b), exhibited flower like morphology with particle size ranging between 100–110 nm. The TEM of PNA-AB, Fig. 2(c), revealed dense spherical particles with sizes ranging between 20–25 nm, whereas the TEM of PLu-AB, Fig. 2(d), showed the formation of spherical clusters with particle size ranging between 25–45 nm. Simialrly, the TEM of PPd-AB, Fig. 2(e), showed clusters of tiny spherical nanoparticles ranging between 10–20 nm.

### UV-visible, Fluorescence emission and singlet oxygen generation kinetics

**Figure 3:**
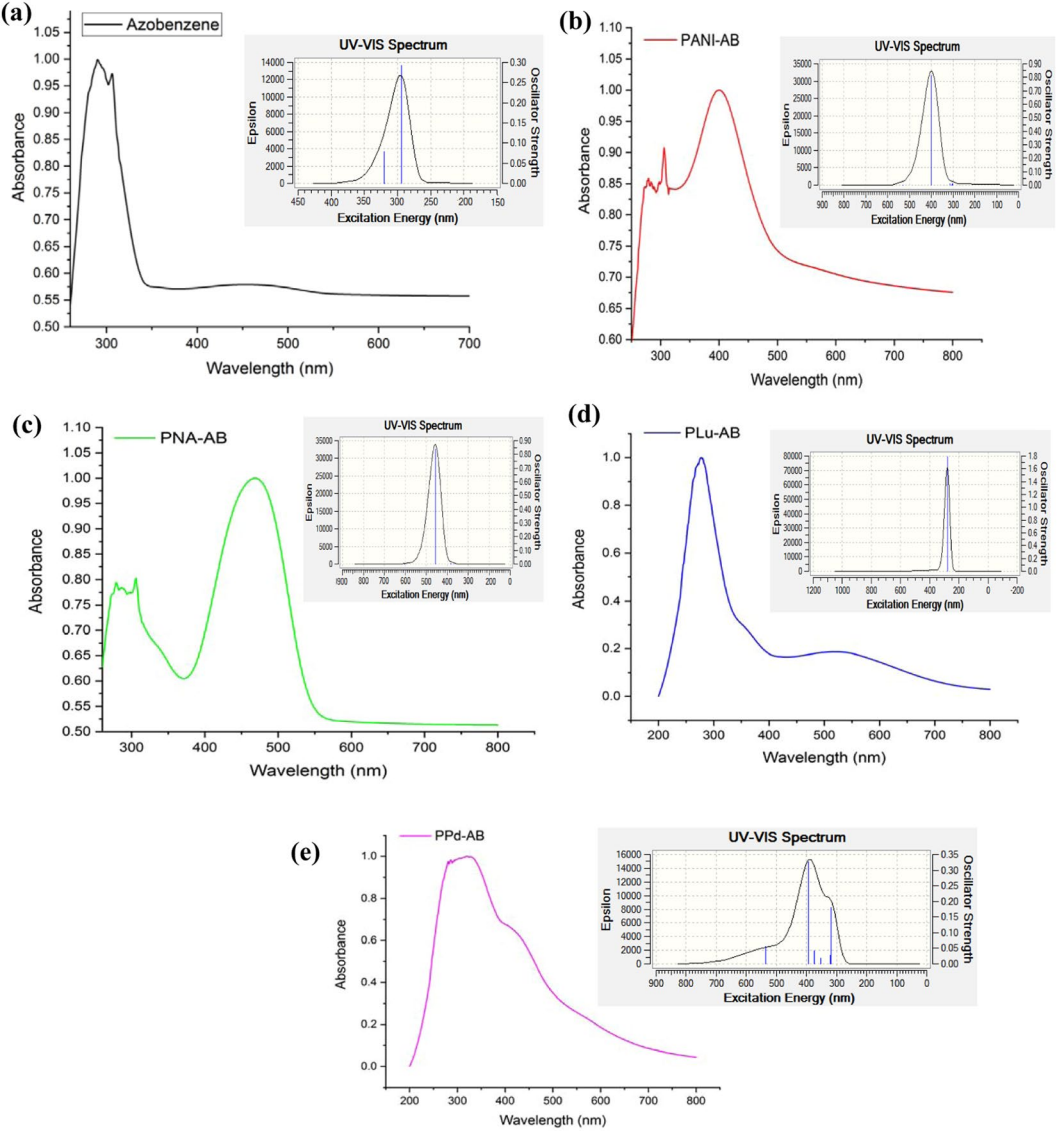
UV-visible spectra of (**a**) AB, (**b**) PANI-AB, (**c**) PNA-AB, (**d**) PLu-AB, (**e**) PPd-AB.

The experimental and DFT/B3LYP calculated UV−vis spectra (shown in inset) are given in Fig. 3(a–e). The UV visible spectrum of AB, Fig. 3(a), showed a prominent peak at 285 nm while the theoretical spectrum revealed the same peak at 290 nm. Azobenzene can exist in E (trans) and Z (cis) forms which can be interconvertible via photochemical as well as thermal processes^30^. Since the Z–E energy difference is approximately in the range 47–48 kJ mol^−1^, the thermodynamically stable form is the E isomer of AB and the conversion of cis isomer to trans isomer is generally irreversible under ambient conditions^30^. The UV spectrum obtained for AB molecule in our case was similar to the one reported for the trans form^31^. Interestingly, the peak corresponding to the existence of trans isomer of AB molecule was found to be pronounced in all the modified polymers which confirmed that upon polymerization, AB existed in its most stable form (trans isomer). The peaks in the UV region noticed in the experimental as well as thoeretical spectra were correlated to the π−π* transition of the benzenoid ring and the oscillator strength values were calculated to be 0.25, 0.26 respectively, Table 1. The UV spectrum of PANI-AB, Fig. 3(b), showed peaks at 310 nm and 400 nm. The later peak was associated with the n−π* transition which was also noted in the theoretical spectrum. The oscillator strength was computed to be 0.83 and 0.85 for the experimental and theoretical peaks respectively. Similarly, the UV spectrum of PNA-AB showed peaks at 280 nm 450 nm and the theoretical spectrum showed a pronounced peak at 460 nm. The oscillator strength values were similar to those observed in PANI-AB and could be correlated to the structural similarity of the two polymers. The polymer PLu-AB, Fig. 3(d), exhibited a prominent peak at 280 nm while a broad hump corresponding to n−π* transition was observed around 520 nm. Likewise, the spectrum of PPd-AB showed an extensive peak around 370 nm which was observed at 400 nm in the theoretical spectrum, Fig. 3(e). The difference of around 30 nm observed in the theoretical and experiment spectrum could be correlated to solvent effect as PPd shows intesne hydrgen bonding with polar solvents. The experimental and theoretical spectra were observed to be in good agreement which confirmed the proposed chmical structure of the polymers.

**Figure 4:**
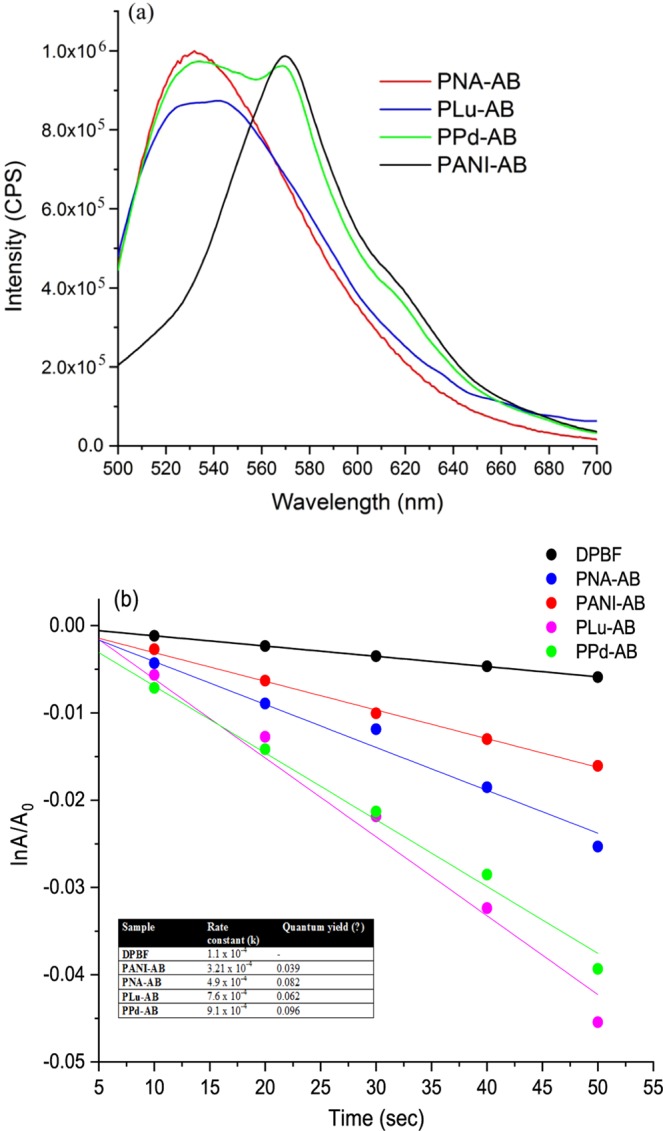
(**a**) Fluorescence spectra of AB based polymers, (**b**) kinetic plot of ^1^O_2_ generation.

Upon excitation at 480 nm, the fluorescence spectra of PANI-AB and PPd-AB exhibited emission maxima at 570 nm while PNA-AB and PLu-AB showed broad emission humps spanning between 500–570 nm corresponding to S_1_→S_o_ transition^22,27^, Fig. 4(a). The quantum yield value was observed to be highest for PPd-AB while it was noticed to be lowest for PANI-AB, Table 1. Upon light irradiation, the polymers were observed to undergo transfer to the excited state and interacted with dissolved oxygen to generate ^1^O_2_. The ^1^O_2_ generation studies were therefore carried out using diphenyl isobenzofuran (DPBF) as quencher^32^. The assays were performed by dissolving the polymers in chloroform (20 µg/mL) and DPBF (6 × 10^−5^ M). The UV spectra were taken upto 50 secs at regular intervals of 10 secs under continuous excitation of laser light (650 nm). The UV absorbance of DPBF at ~410 nm was plotted as a function of the irradiation time (given in Supplementary Information as Fig. S5(a–d)). The studies showed that pure DPBF showed negligible changes under the laser light irradiation. The polymers however showed a prominent decrease in the absorbance value at ~410 nm. A plot of optical absorbance at 410 nm as a function of irradiation time was consistent with first order kinetics, Fig. 4(b). The kinetic plot revealed that PPd-AB, Fig. 4(b), exhibited the highest k value of 9.1 × 10^−4^ s^−1^ while PANI-AB showed a value of 3.2 × 10^−4^ s^−1^. The ^1^O_2_ generation quantum yield values for the polymers were determined by reported method^32^. The ^1^O_2_ quantum yield value for the PPd-AB was found to be 0.091. It can therefore be concluded that the polymers can act as effective photosensitizers and can be utilized in photodynamic therapy.

### Studies on antileishmanial activity

**Figure 5:**
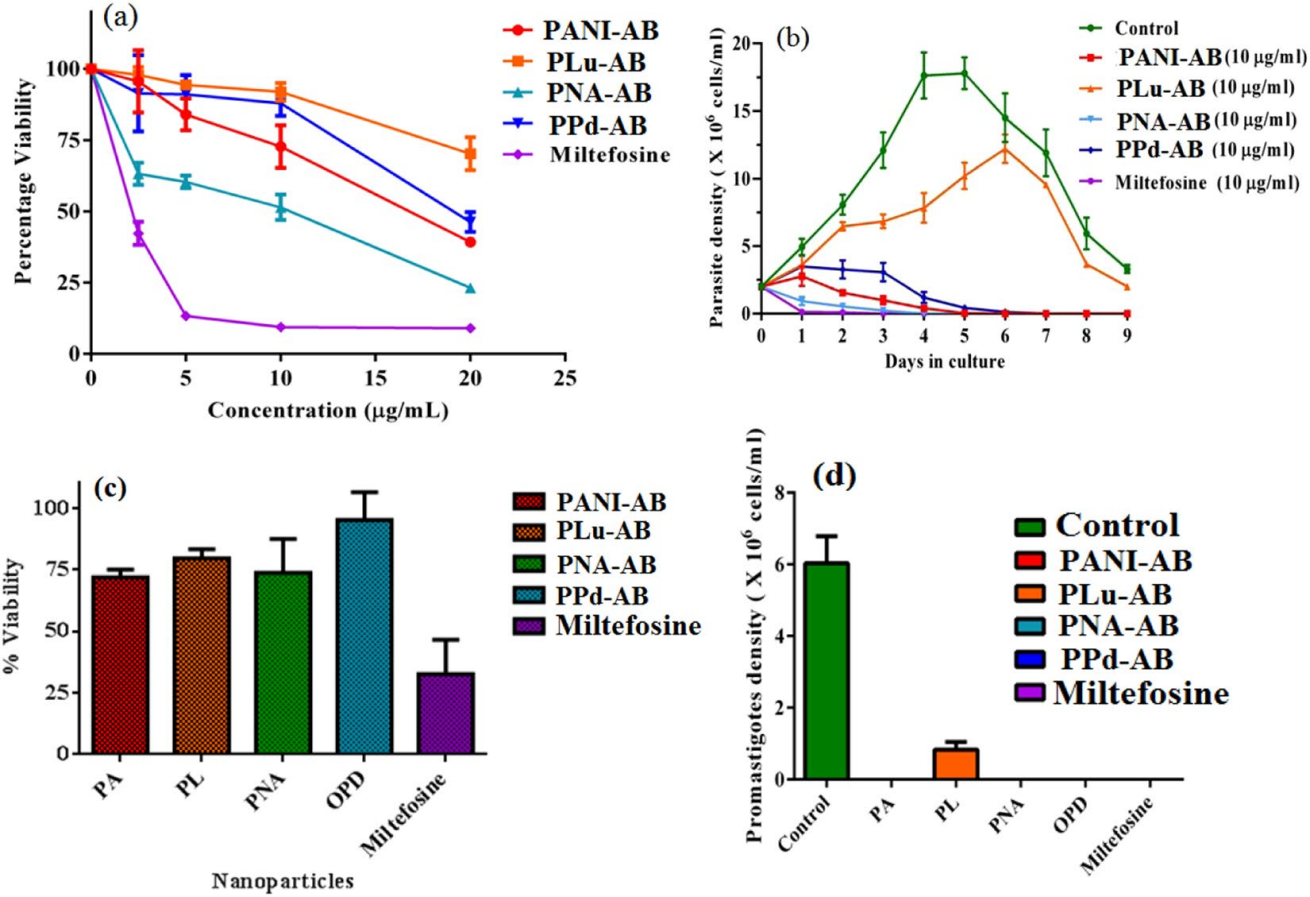
(**a**) Leishmanicidal activity of nanoparticles at different concentration: 2 × 10^6^ promastigotes were cultured in presence of PANI-AB, PNA-AB, PLu-AB, PPd-AB and Miltefosine at 2.5, 5, 10 and 20 µg/mL for 48 h, (**b**) Effect of nanoparticles on promastigote growth: Parasites cultivated at 25 °C in M199 supplemented with 10% of fetal bovine serum (control) or treated with 10 µg/mL PANI-AB, PNA-AB, PLu-AB, PPd-AB and Miltefosine monitored daily for nine days. (Counts were performed using hemocytometer), (**c**) Cytotoxic activity of nanoparticles at 10 μg/mL for 48 h, (**d**) Growth Reversibility assay: Parasites treated with 10 µg/mL of PANI-AB, PNA-AB, PLu-AB, PPd-AB and Miltefosine for nine days. The cells were counted after 48 h. Bars represent means and standard errors obtained from three independent experiments.

**Figure 6:**
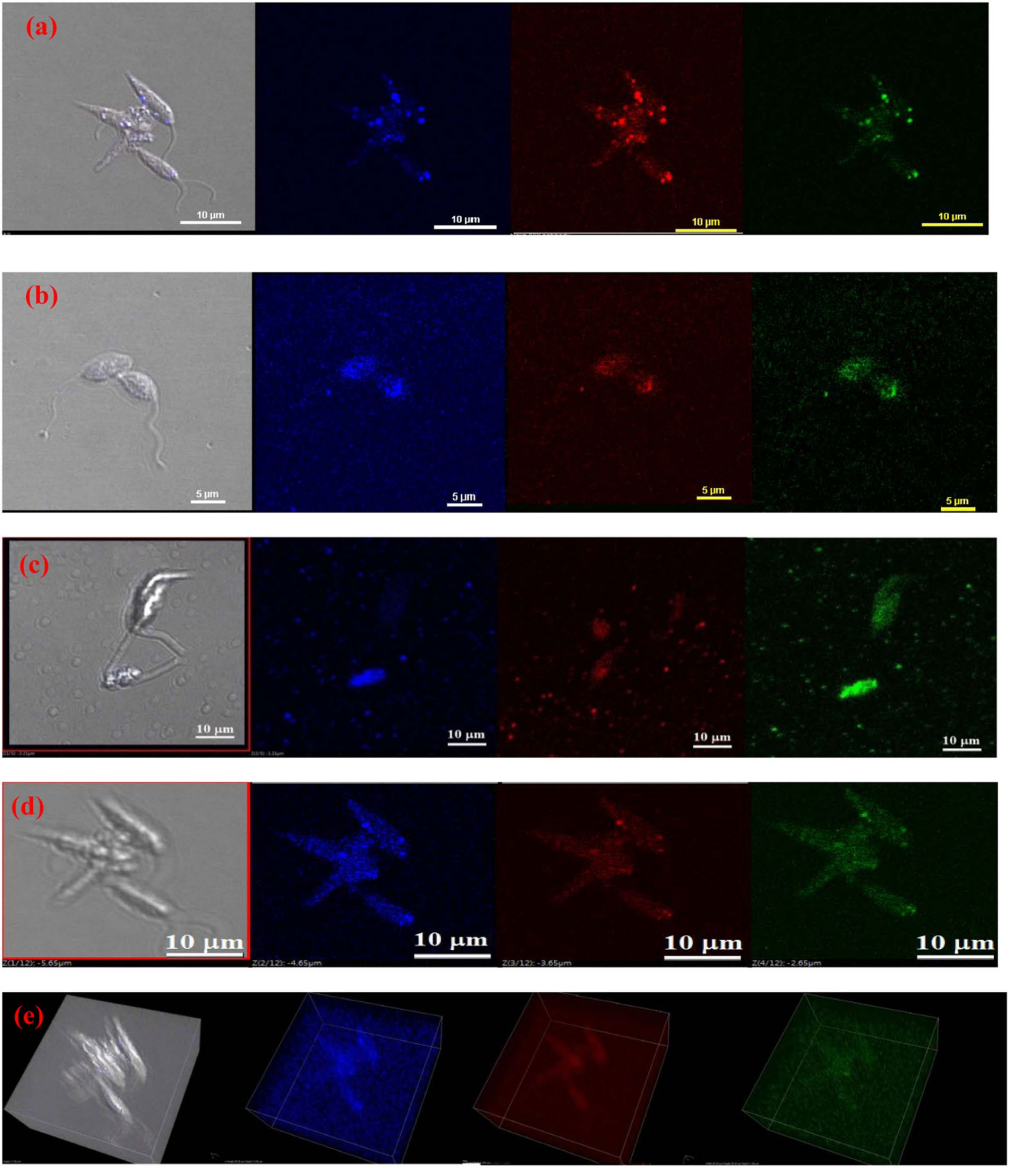
Live Cell imaging of (**a**) PANI-AB, (**b**) PNA-AB, (**c**) PLu-AB, (**d**) PPd-AB, (**e**) 3D image of leishmania treated with PNA-AB.

*Leishmania* evades immune host immune system by dampening critical signalling pathways which are required for parasite clearance^5,6^. In this regard, the development of less toxic nanoparticles could also be helpful in providing therapeutic benefits^7^. The potential antileishmanial activity of PANI-AB, PNA-AB, PLu-AB and PPd-AB was explored and the polymers were found to be non-toxic as compared to miltefosine, the standard antileishmanial drug at equivalent concentrations. The polymers showed *in-vitro* antileishmanial activity up to 48 h, Fig. 5(a). The IC_50_ value for miltefosine is 6.9 µg/ml. The IC_50_ values of PANI-AB, PLu-AB, PNA-AB and PPd-AB were evaluated as 10.7 µg/ml, 23.9 µg/ml, 12.3 µg/ml, 19.2 µg/ml respectively. These polymers were further tested for promastigote growth kinetics and both PANI-AB as well as PNA-AB were found to be more effective against promastigotes in comparision to PPd-AB. PLu-AB was the least effective among all the tested polymers, Fig. 5(b). The cytotoxicity assays on THP-1 cell line derived, human macrophages showed that the polymers could be safely used at a concentration of 10 µg/ml as compared to miltefosine, Fig. 6(c). Furthermore, the parasites were washed after the treatment and monitored for growth reversibility, and it was found that promastigotes treated with PANI-AB, PNA-AB and PPd-AB failed to return to a viable state, while untreated promastigote returned back to late log phase. However, PLu-AB treated promastigotes did not revert back to normal morphology after 96 h, Fig. 5(d). The possibility of using these fluorescent polymers as effective imaging agents for *Leishmania* parasites was investigated by carrying out live cell imaging experiments by treating *Leishmania* parasites with the polymers as depicted in Fig. 6(a–d).

It was observed that PANI-AB, Fig. 6(a), exhibited intense green and red emission as compared to blue emission while the emission intensity of PLU-AB, Fig. 6(b), was observed to be lower. This was due to higher extent of penetration of PANI-AB into the promastigote as compared to PLu-AB. Similarly, in case of PPd-AB, Fig. 6(c), the intensity of green emission was observed to be higher as compared to blue emission while for PNA-AB, Fig. 6(d), the extent of penetration of the polymer was found to be higher under different lasers. This was further confirmed by the 3D image, Fig. 6(e). The images clearly revealed that the polymer binds to the plasma membrane before trafficking via endocytic pathway. The polymers can therefore be effectively used to stain and image *Leishmania* promastigotes to monitor their growth as well as apoptosis.

## Conclusion

Azo modified polymers were synthesized and confirmed by experimental as well as theoretical FTIR, UV-visible and ^1^H-NMR studies. The polymers exhibited intense fluorescence in the IR region and the intensity of emission was governed by the extent of conjugation attained. Antileishmanial activities showed that the polymers PANI-AB, PNA-AB, and PPd-AB have potent *in-vitro* antileishmanial activity. Cytotoxicity studies on human macrophages revealed that the polymers were potentially safe at doses equivalent to miltefosine. The live cell imaging studies of PNA-AB stained *Leishmania* promastigotes showed intense emission in green as well as red regions. As these polymers were found to be least-toxic, they can be safely used as fluorescent markers to label and target parasite in *Leishmania* infected patients.

## Supplementary information

supplementary information.
