# Supplementary material for: Experimental and Theoretical Studies of Novel Azo Benzene Functionalized Conjugated Polymers: In-vitro Antileishmanial Activity and Bioimaging
Source: Sci Rep. 2020 Jan 9;10:57. doi: 10.1038/s41598-019-56975-x (PMC6952527; doi:10.1038/s41598-019-56975-x)
Supplement: Supplementary file 1 — supplementary information. [file 41598_2019_56975_MOESM1_ESM.docx]

**Supporting Information**

Experimental and Theoretical Studies of Novel Azo Benzene Functionalized Conjugated Polymers: Invitro Antileishmanial Activity and Bioimaging

Neetika Singh^a^, Mohd. Arish^b^, Prabhat Kumar^c^ Abdur Rub^b *^, Ufana Riaz^a*^,

^a^ Materials Research Laboratory Department of Chemistry, Jamia Millia Islamia, New Delhi-110025, India, ^b^ Department of Biotechnology, Jamia Millia Islamia, New Delhi-110025, India, ^c^Advance Instrumentation Research Facility, Jawaharlal Nehru University, New Delhi- 110067, India, ^*^Corresponding author email: [ufana2002@yahoo.co.in](mailto:ufana2002@yahoo.co.in), [arub@jmi.ac.in](mailto:arub@jmi.ac.in)


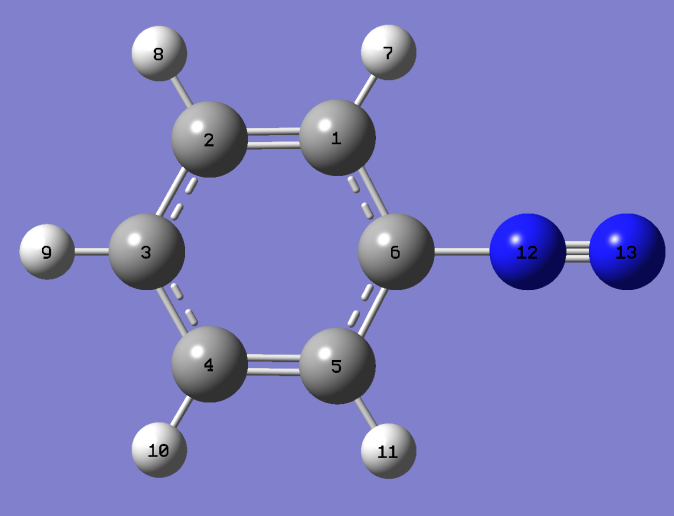


**(a)**

**(b)**

**
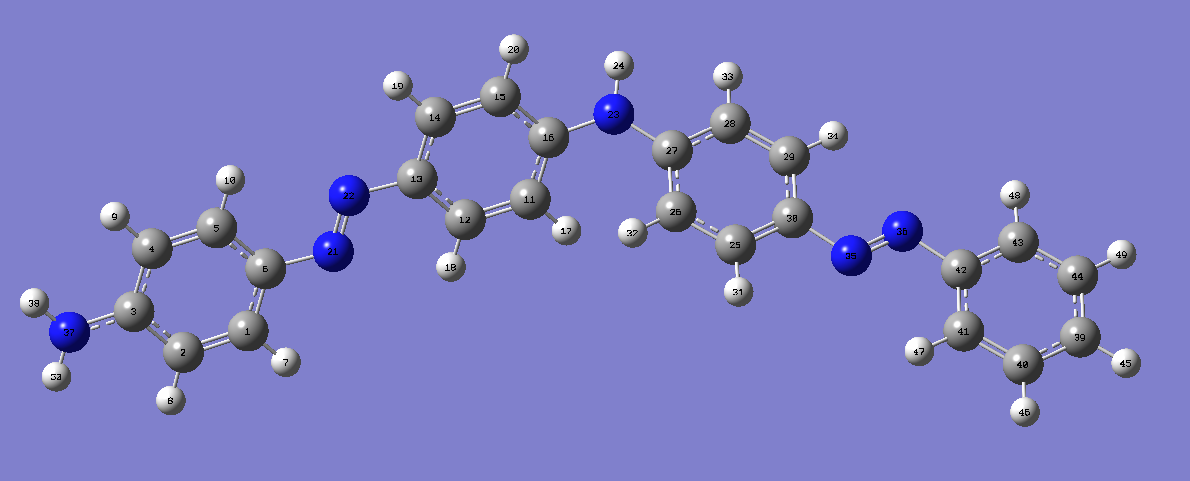
**

**
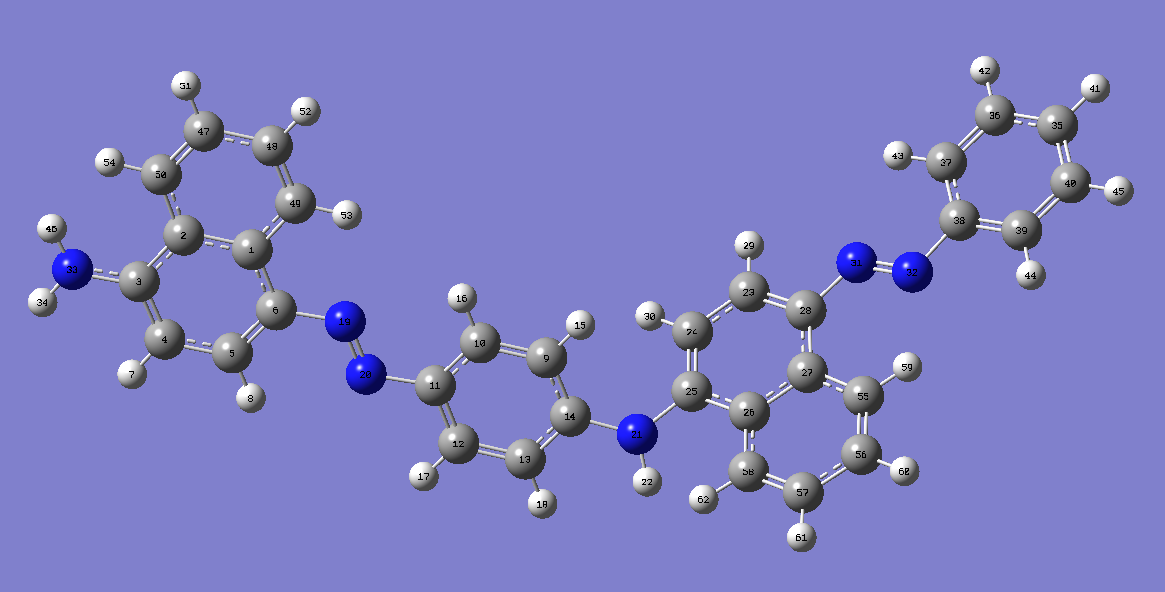
**

**(c)**

**
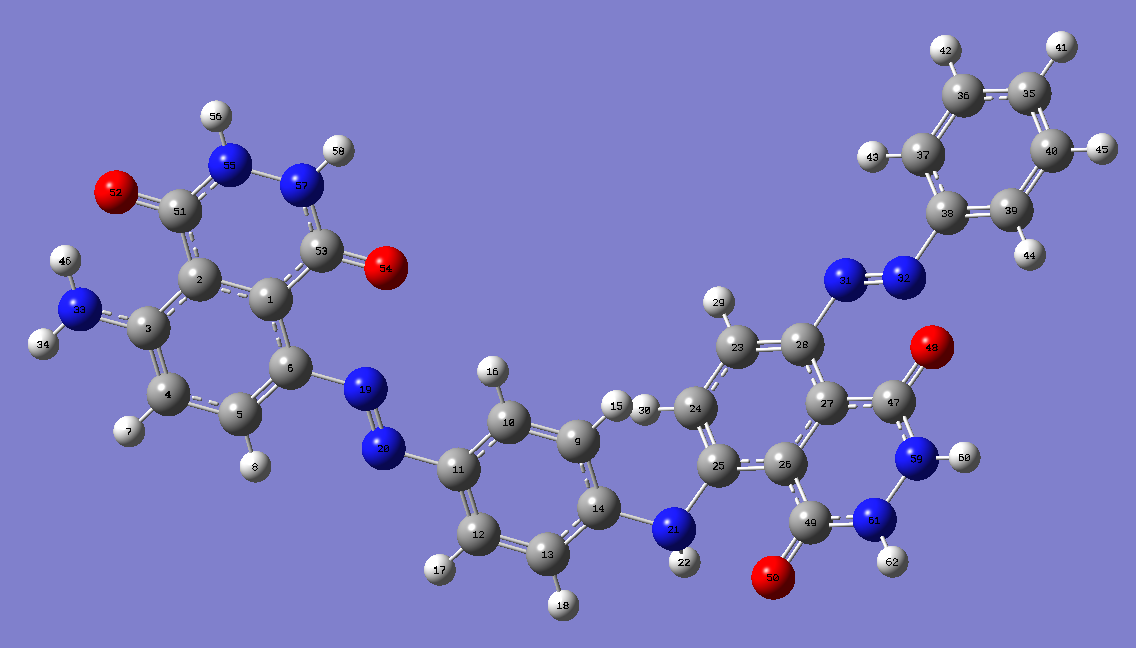
**

**(d)**

**
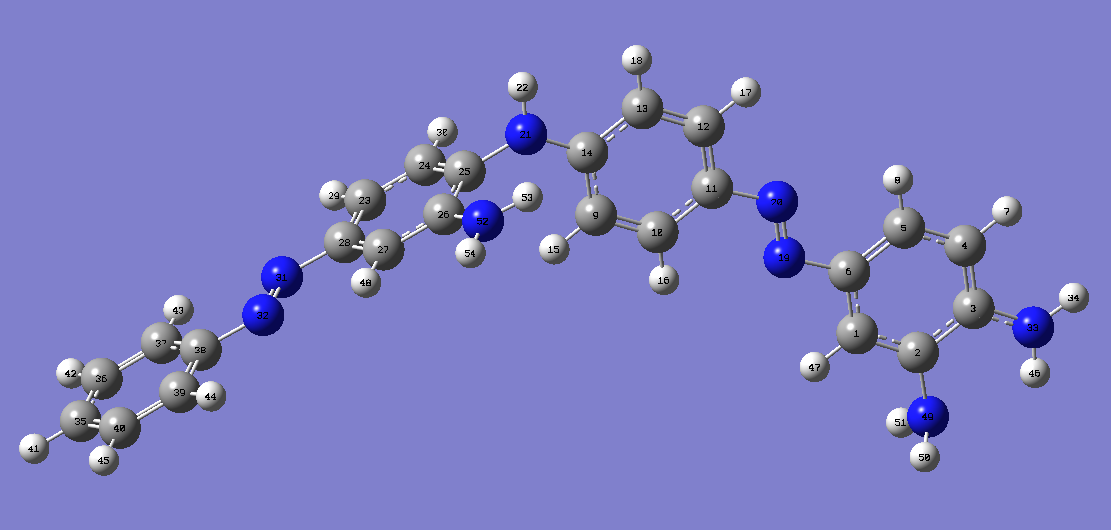
**

**(e)**

**Figure S1 Optimized geometries of (a) azo benzene, (b) PANI-AB, (c) PNA-AB, (d) PLu-AB, (e) PPd-AB**


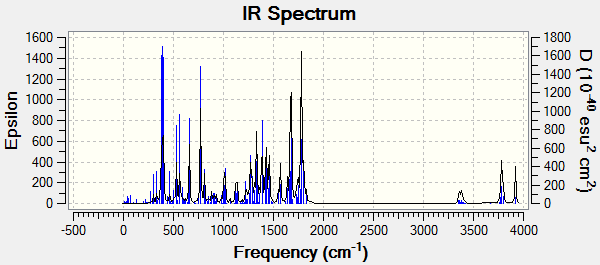

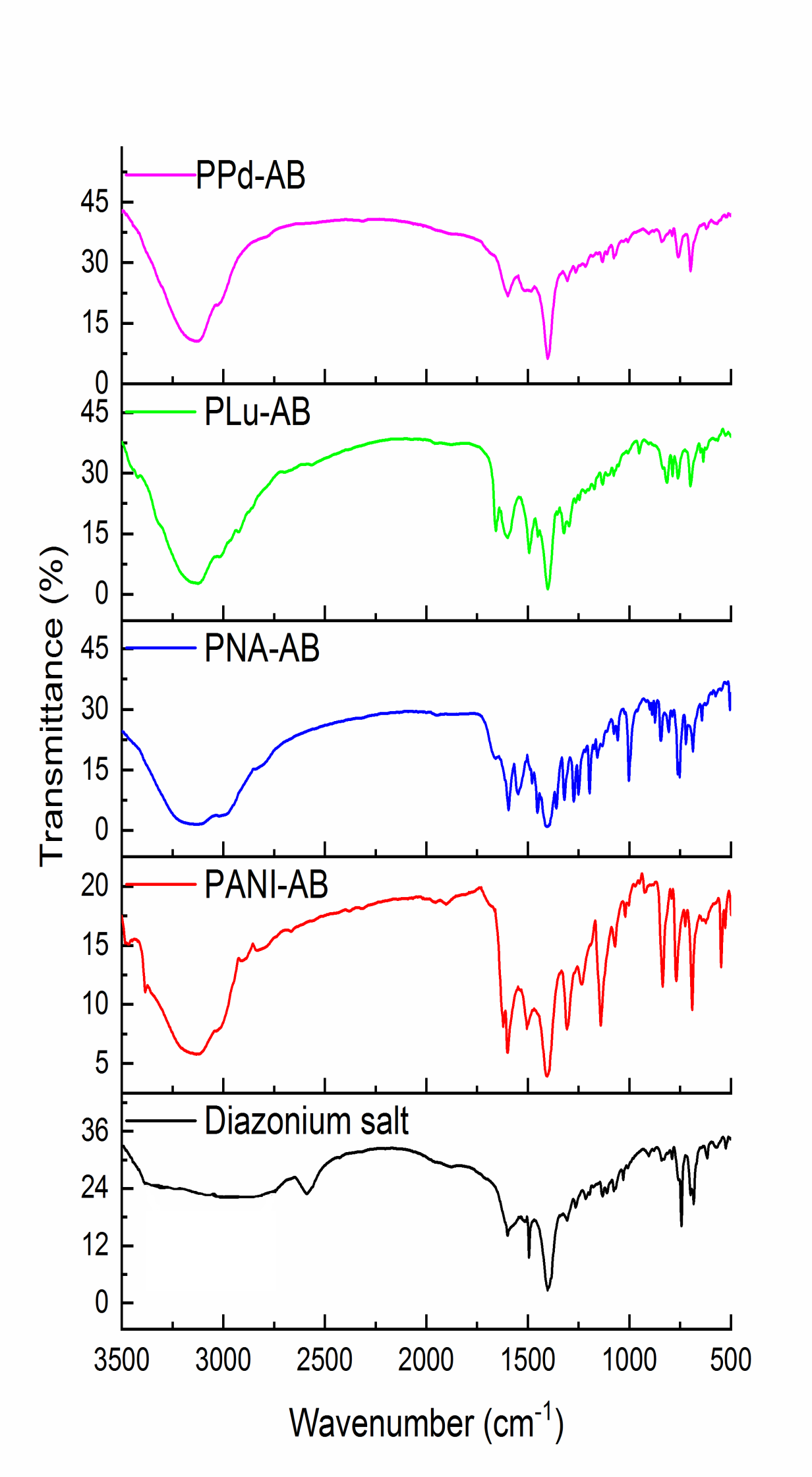


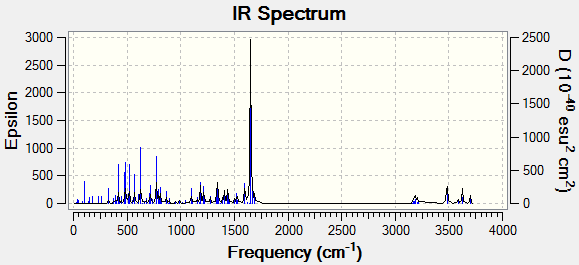


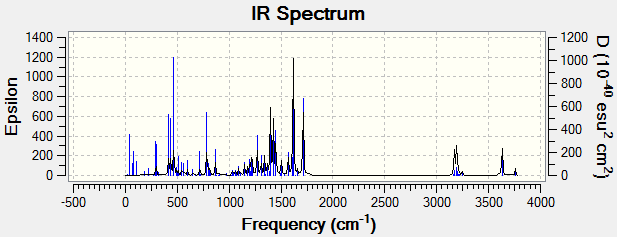


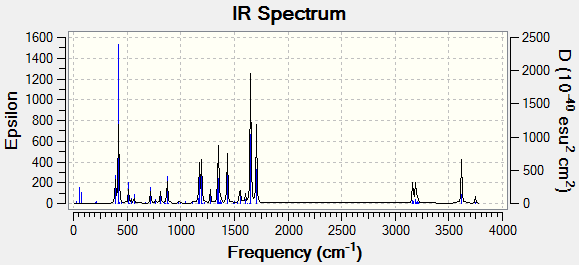


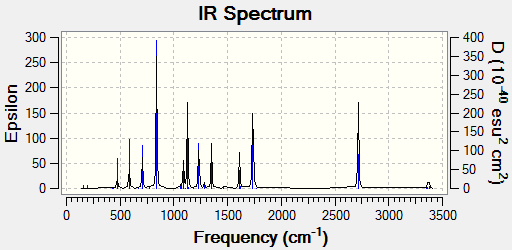


**Figure S2 IR spectra of azo benzene and conjugated polymers of PANI, PNA, PLu and PPd**

**(a)**


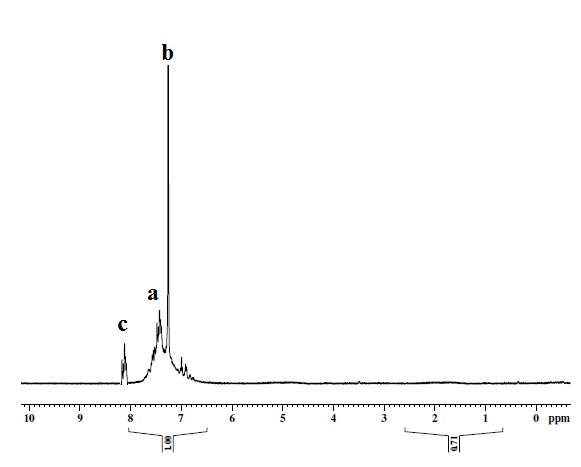

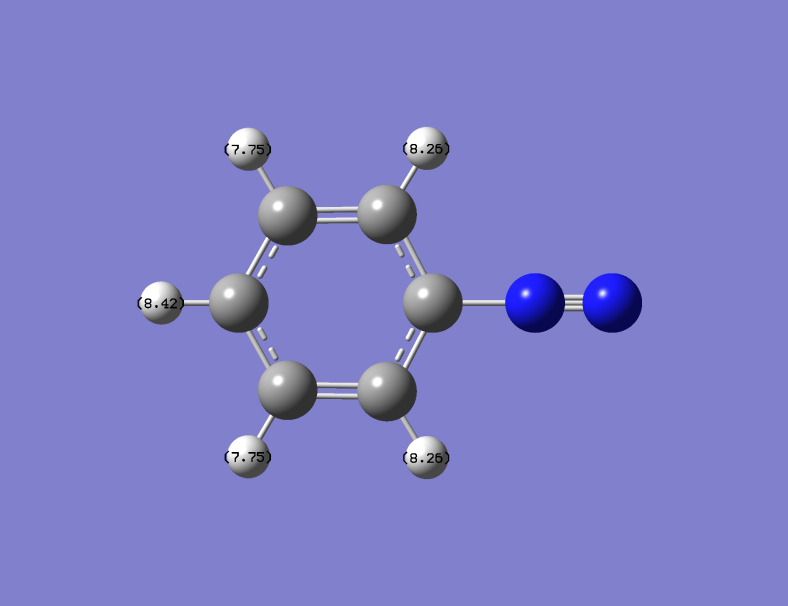

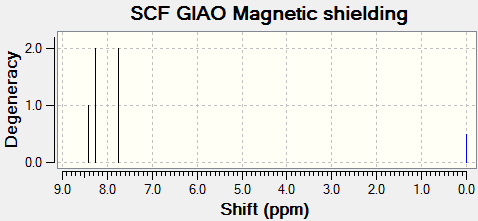


**(b)**


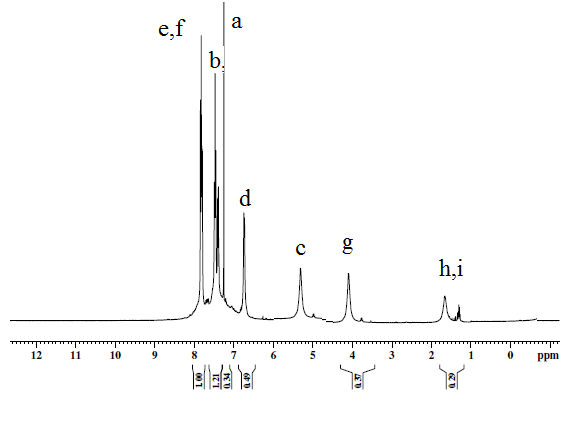

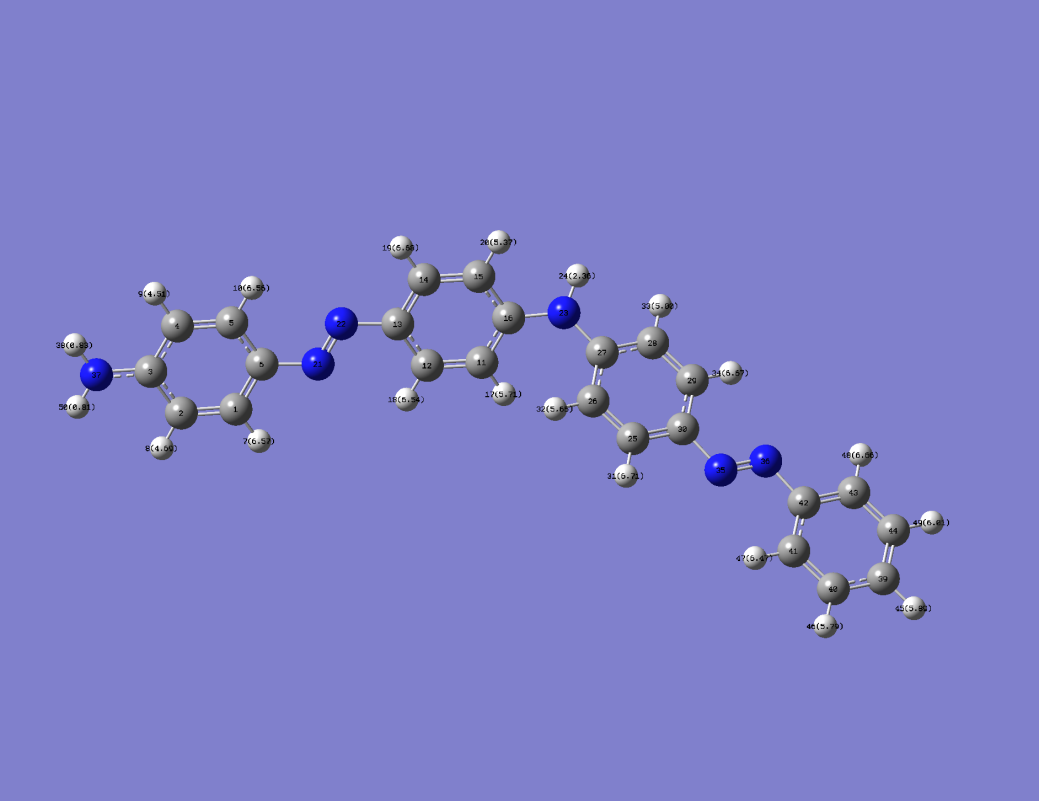

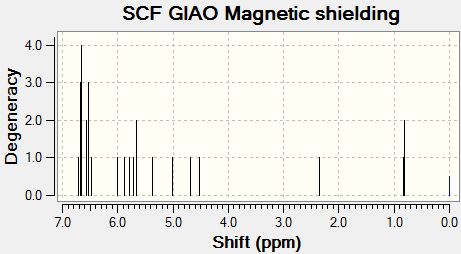


**(c)**

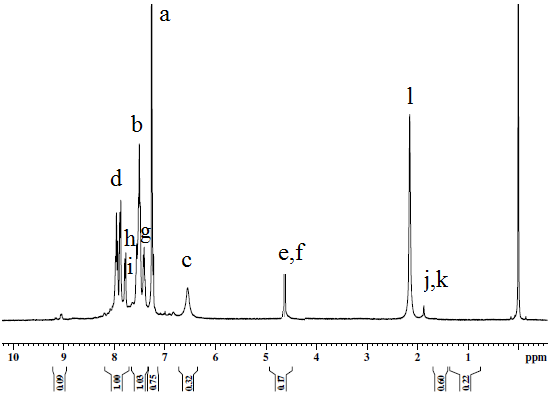


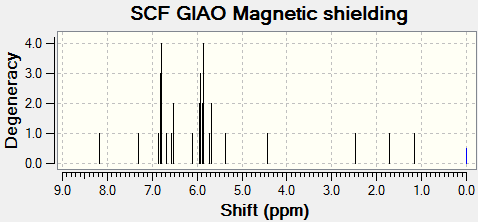


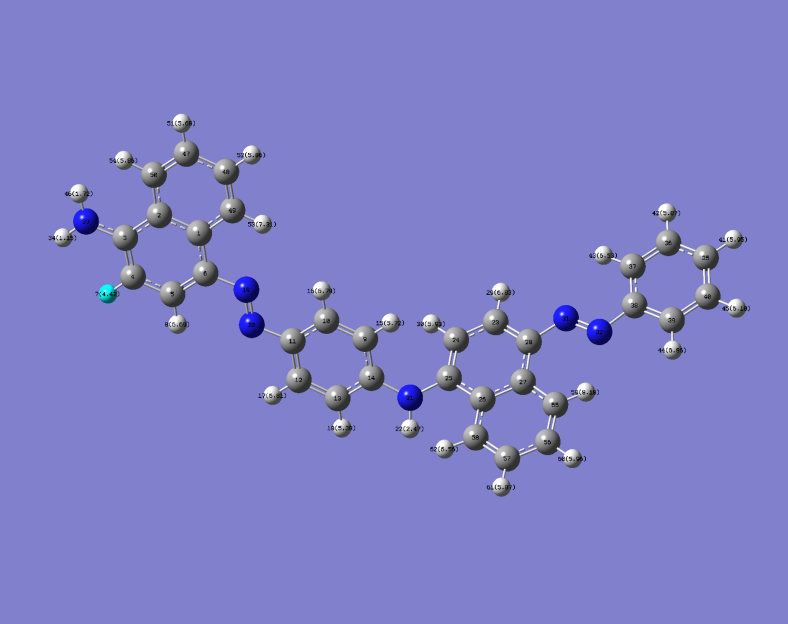


**(d)**

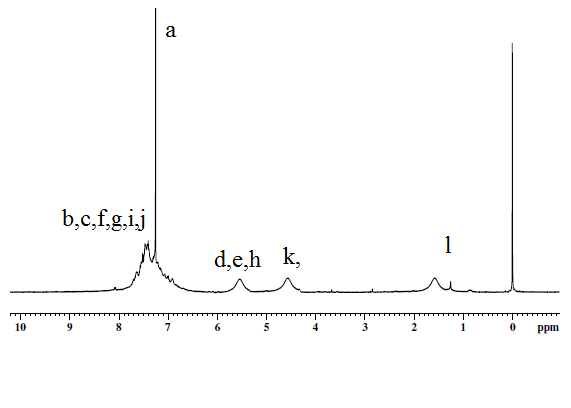


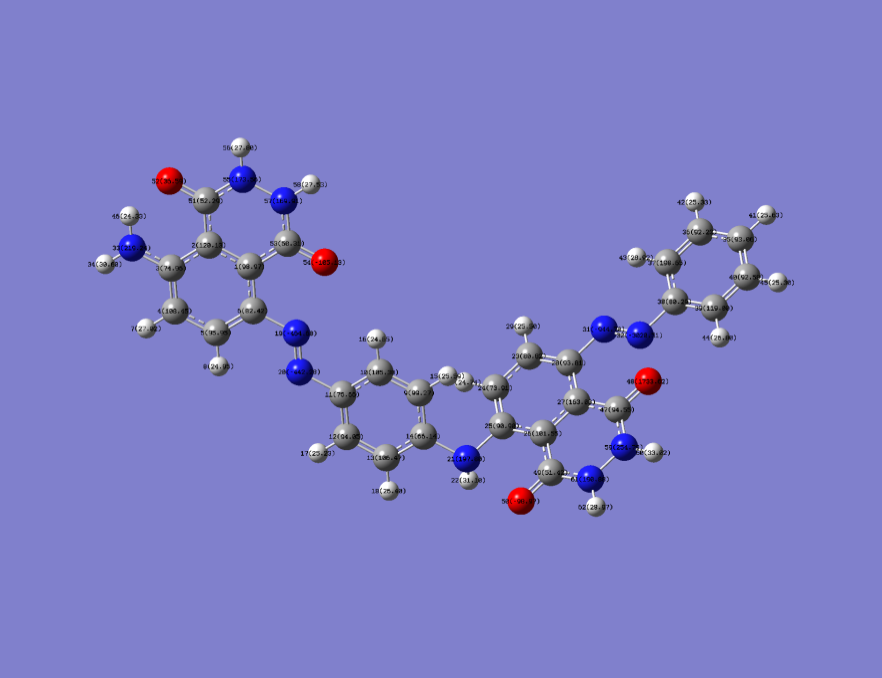

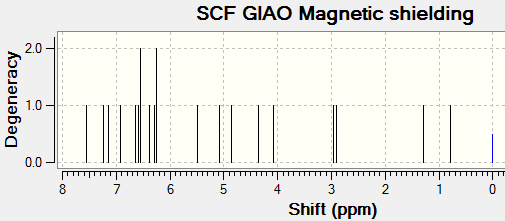


**(e)**

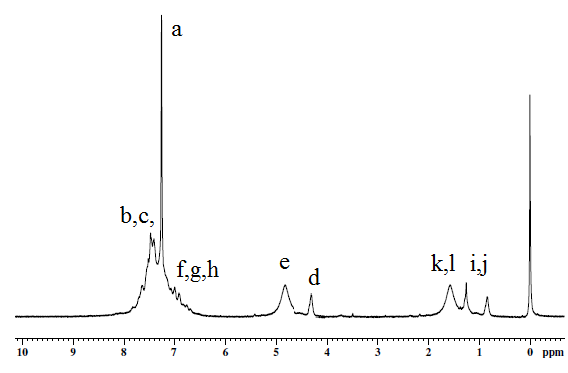


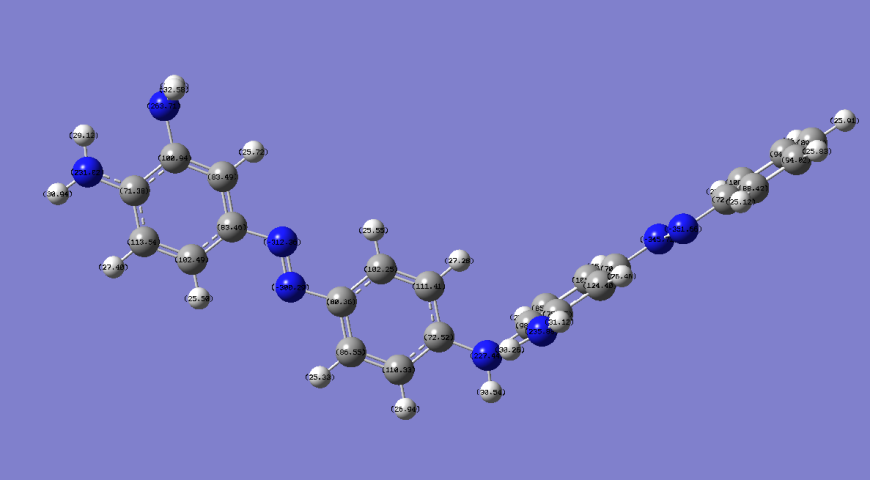

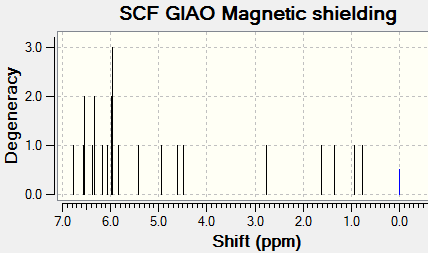


**Figure S3 ^1^H-NMR spectra of (a) azobenzene, (b) PANI-AB, (c) PNA-AB , (d) PLu-AB and (e) PPd-AB**

**Figure S4 XRD profiles of diazonium salt and its polymers with PANI, PNA, PLu and PPd**

**Figure S5 UV spectra of DPBF in the presence of (a) PANI-AB, (b) PNA-AB,**

**(c) PLu-AB, (d) PPd-AB**
